# Association of oxidative balance score with hyperuricemia and gout: NHANES 2009-2018

**DOI:** 10.3389/fendo.2024.1402369

**Published:** 2024-11-04

**Authors:** Yiting He, Xiaojing Chen, Zeming Ma, Jingsa Wang, Kun Lin

**Affiliations:** ^1^ College of Medicine, Shantou University, Shantou, China; ^2^ Department of Endocrinology and Metabolism, First Affiliated Hospital of Shantou University Medical College, Shantou, China

**Keywords:** oxidative balance score, hyperuricemia, gout, NHANES, antioxidants, oxidative stress

## Abstract

**Introduction:**

Oxidative stress plays a crucial role in the development and progression of hyperuricemia/gout. This study aims to explore the relationship between the Oxidative Balance Score (OBS) and hyperuricemia/gout.

**Methods:**

The study utilized complete data from adult participants in the National Health and Nutrition Examination Survey (NHANES) spanning from 2009 to 2018. OBS, composed of scores for 20 dietary and lifestyle factors, served as the exposure variable. Multivariable linear regression model was applied to evaluate the association between OBS and uric acid (UA). Multivariable logistic regression, subgroup analyses, and restricted cubic spline (RCS) regression were conducted to explore the relationship between OBS and hyperuricemia/gout.

**Results:**

A total of 18,998 participants were included. In the fully adjusted model, compared to the lowest quartile, the highest quartiles of OBS, dietary OBS, and lifestyle OBS were negatively correlated with UA (β=-0.31 (-0.36,-0.25), β=-0.18 (-0.24,-0.12), and β=-0.64 (-0.69,-0.59), respectively) and hyperuricemia (OR=0.63 (0.55,0.71), OR=0.76 (0.67,0.86), OR=0.37 (0.33,0.42), respectively). Moreover, the highest quartiles of OBS and lifestyle OBS exhibited a negative correlation with gout (OR=0.72(0.58,0.91), OR=0.54 (0.43,0.67), respectively). Subgroup analyses revealed differences in the negative association between OBS and hyperuricemia concerning hypertension (p for interaction =0.002) and diabetes (p for interaction= 0.004), while gender-related disparities were observed in the negative association between OBS and gout (p for interaction =0.008). RCS analysis demonstrated a linear negative association between hyperuricemia and OBS (p for non-linearity >0.05), while gout exhibited a non-linear negative association (p for non-linearity<0.05).

**Conclusion:**

The study found that a higher OBS was associated with a decreased risk of developing hyperuricemia/gout, underscoring its potential in the prevention and management of these conditions.

## 1 Introduction

Uric acid (UA), a terminal product of purine analog metabolism, plays a crucial role in the development of hyperuricemia (1). Hyperuricemia is characterized by excessive production, arising from increased endogenous purine catabolism and excessive exogenous purine intake, or by impaired plasma UA excretion (2). The manifestation of hyperuricemia occurs when UA levels surpass a defined threshold, signifying the onset of a chronic metabolic disorder. Prolonged deposition of substantial UA amounts in joints and tissues eventually progresses into gout (3), affecting individuals of all ages and genders. Gout can manifest as recurrent acute attacks accompanied by joint damage and bone erosion, leading to functional impairment in severe cases (4). Additionally, hyperuricemia emerges as an independent risk factor for various systemic diseases, including cardiovascular diseases (5), hypertension (6), diabetes (7), and chronic kidney disease (CKD) (8).

Oxidative stress is usually defined as an imbalance between reactive oxygen species (ROS) and antioxidants in our body, recognized as a leading cause of cell damage and disease development. A large body of evidence has demonstrated that oxidative stress plays a crucial role in the development and progression of hyperuricemia/gout (4, 9). The oxidative balance score (OBS) is a comprehensive indicator containing 20 distinct dietary and lifestyle components, offering a means to quantify exposure to antioxidants and pro-oxidants in diet and lifestyle, therefore reflecting the overall burden of oxidative stress (10). Generally, a higher OBS is indicative of a preference for antioxidants over pro-oxidants (11). Numerous epidemiological studies have found inverse associations between OBS and various inflammation-related diseases, including CKD (12), cardiovascular disease (13), osteoarthritis (14), and type 2 diabetes (15). Nevertheless, the existing research has yet to explore the association between OBS and hyperuricemia/gout.

Recognizing the significance of OBS and implementing timely interventions may be of great value in preventing the progression of hyperuricemia and facilitating the remission of gout. To address this knowledge gap, we employ the well-established OBS in a nationally representative survey, the National Health and Nutrition Examination Survey (NHANES), to investigate its impact on hyperuricemia and gout for the first time.

## 2 Method

### 2.1 Source of data and study population

**Figure 1:**
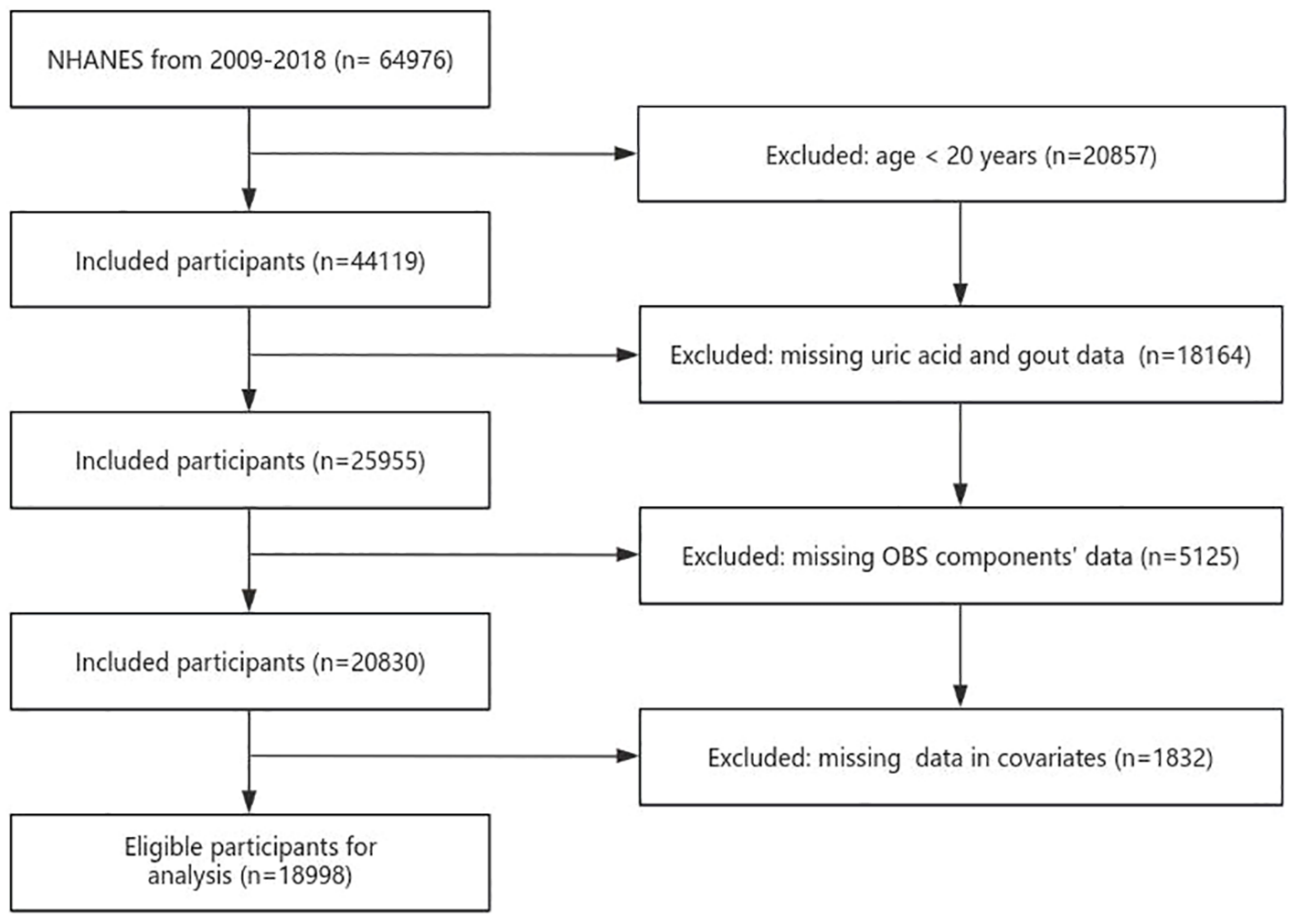
Flowchart of the sample selection from NHANES 2009–2018.

The NHANES conducted a national cross-sectional study to assess the health and nutrition status of both adults and children within the U.S. population. Employing a “stratified multistage probability sampling” method, the study gathered information through interviews, examinations, dietary questionnaires, and laboratory measurements. A total of 18998 participants were chosen from 2009 to 2018. Exclusion criteria were as follows: age of participants was <20 years, participants without serum UA and gout data, participants without OBS components’ data, and variables with missing values (**Figure 1**). All participants provided signed written informed consent, and the study conformed to ethical standards.

### 2.2 Calculation of OBS

The construction of OBS in this study was based on both prior research findings and experiential insights (16). The OBS incorporated 16 nutrients and 4 lifestyle factors. These 20 components were further classified into pro-oxidants (total fat, iron, alcohol intake, BMI, and cotinine) and antioxidants (dietary fiber, β-carotene, vitamin B2, niacin, vitamin B6, total folate, vitamin B12, vitamin C, vitamin E, calcium, magnesium, zinc, copper, selenium, and physical activity). The mean of the two 24-hour for diet and dietary supplements represented dietary and dietary supplement intake, and the intake of each nutrient was the sum of diet and dietary supplements (if available). Due to the absence of supplementation data for vitamin E and β-carotene from dietary supplements in NHANES, only dietary data were used for these two nutrients. Following Zhang et al. (16)’s method for calculating OBS, alcohol consumption was stratified into three groups, heavy drinkers (≥15 g/d for women and ≥30 g/d for men), non-heavy drinkers (0-15 g/d for women and 0-30 g/d for men), and nondrinkers, who were assigned 0, 1, and 2 points, respectively. Subsequently, the other components were categorized based on gender and further divided into three groups according to their tertile distributions. Antioxidants were assigned scores of 0-2 in groups 1-3, while pro-oxidants were assigned scores of 2-0 in groups 1-3, respectively (**Supplementary Table 1**). The total OBS score was the sum of scores for each component, where a higher OBS indicated a greater predominance of antioxidant exposure.

### 2.3 Evaluation of hyperuricemia and gout

Hyperuricemia was defined as a level of serum UA that was ≥416 mmol/L (7 mg/dL) in men and ≥357 mmol/L (6 mg/dL) in women (17). Gout was defined based on self-reported diagnosis by physicians, ascertained through the following question: “Has a doctor or other health professional ever told you that you have gout?” (17).

### 2.4 Covariates

Based on available literature and clinical considerations, we identified the following covariates considered potential confounders in the associations between OBS and hyperuricemia/gout. Standardized household interviews provided demographic characteristics, including age, gender (male and female), race (non-Hispanic white, non-Hispanic black, Mexican American, and other races), educational level(below high school, high school, and above high school), marital status (never married, married or lived with a partner, and others), family poverty income ratio (PIR) and total energy intake. Hypertension, diabetes, hyperlipidemia, and CKD are recognized as significant risk factors for hyperuricemia. Consequently, these diseases were integrated into the analysis. The diagnostic criteria for hypertension included an average systolic blood pressure ≥140 mmHg or average diastolic blood pressure ≥90 mmHg after at least three measurement, the use of antihypertensive drugs, and a physician-reported diagnosis of hypertension (18). Diabetes was diagnosed based on glycated hemoglobin (HbA1c) >6.5%, fasting glucose ≥7.0 mmol/L, use of diabetes medication or insulin and a previous diagnosis of diabetes by a physician (19). Hyperlipidemia was determined by total cholesterol levels ≥200 mg/dL, triglyceride levels ≥150 mg/dl, high-density lipoprotein-cholesterol ≤40 mg/dL in men and ≤50 mg/dL in women, and low-density lipoprotein-cholesterol ≥130 mg/dL (20). An albumin-to-creatinine ratio (ACR) ≥30 mg/g (3 mg/mmol) and estimated glomerular filtration rate (eGFR) <60 ml/min/1.73 m^2^ were adopted as diagnostic criteria for CKD (21). we utilized the Chronic Kidney Disease Epidemiology Collaboration formulate to evaluate eGFR (22).

### 2.5 Statistical analysis

OBS was treated as a continuous variable. OBS group (quartile conversion) was considered a categorical variable. Continuous variables are presented as median (interquartile range (IQR)), and categorical variables are presented as frequency (percentage). Baseline characteristics were compared using the chi-square test for categorical variables and the Kruskal-Wallis test for continuous variables. To evaluate the association between OBS and UA, coefficient (β) and 95% confidence intervals (CI) were calculated using multivariable linear regression models. To explore the relationship between OBS and hyperuricemia/gout, odds ratio (OR) and 95% CI were calculated using multivariable logistic regression. The crude model was not adjusted for any covariates. Model 1 was adjusted for age, gender, and race. Model 2 was further adjusted for education level, family PIR, marital status, hypertension, diabetes, hyperlipidemia, CKD and energy. Heterogeneity between OBS (as a continuous variable) and hyperuricemia/gout was assessed through interaction and subgroup analyses for the following variables, including gender, age groups, education, race, marital status, family PIR, hypertension, hyperlipidemia, diabetes and CKD. Restricted cubic spline (RCS) analysis with four knots was applied to evaluate non-linear associations between OBS/dietary OBS/lifestyle OBS and hyperuricemia/gout risk. All statistical analyses were performed using R Statistical Software (Version 4.2.2, http://www.R-project.org, The R Foundation) and the Free Statistics analysis platform (Version 1.9, Beijing, China). Alpha was set at <0.05 for statistical significance, and all analyses were two-sided, considering a two-sided *p*-value <0.05 as statistically significant.

## 3 Results

### 3.1 Baseline characteristics

Baseline characteristics of individuals grouped by OBS quartiles are shown in **Table 1**. The median age of subjects was 49 years, with 52.3% being female. The majority of participants were non-Hispanic white (43.5%), and the overall prevalence of hyperuricemia and gout in the entire U.S. population was 20.1% and 4.7%, respectively. Compared to the lowest OBS quartile (Q1), individuals in the highest OBS quartile (Q4) had higher age, higher education levels, greater wealth, higher total energy intake, lower UA levels, were married or partnered, and were non-Hispanic white or of other races. The prevalence of hyperuricemia and gout, along with their comorbidities, including hypertension, diabetes, hyperlipidemia, and CKD, gradually decreased as OBS increased.

**Table 1:** The baseline characteristics by quartiles of the OBS: National Health and Nutrition Examination.

Characteristic	Total	Q1	Q2	Q3	Q4	*P* value
	n = 18998	n = 4983	n = 4614	n = 5288	n = 4113	
Gender, n (%)						0.259
Male	9071 (47.7)	2325 (46.7)	2241 (48.6)	2521 (47.7)	1984 (48.2)	
Female	9927 (52.3)	2658 (53.3)	2373 (51.4)	2767 (52.3)	2129 (51.8)	
Age, Median (IQR)	49.0 (35.0, 63.0)	49.0 (34.0, 63.0)	47.0 (33.0, 62.0)	49.0 (35.0, 63.0)	53.0 (38.0, 66.0)	< 0.001
Race, n (%)						< 0.001
Non-Hispanic White	8260 (43.5)	1866 (37.4)	1889 (40.9)	2352 (44.5)	2153 (52.3)	
Non-Hispanic Black	3918 (20.6)	1525 (30.6)	970 (21)	898 (17)	525 (12.8)	
Mexican American	2625 (13.8)	652 (13.1)	694 (15)	800 (15.1)	479 (11.6)	
Others	4195 (22.1)	940 (18.9)	1061 (23)	1238 (23.4)	956 (23.2)	
Education level, n (%)						< 0.001
< High school	3891 (20.5)	1407 (28.2)	1026 (22.2)	937 (17.7)	521 (12.7)	
High school	4267 (22.5)	1367 (27.4)	1074 (23.3)	1103 (20.9)	723 (17.6)	
> High school	10840 (57.1)	2209 (44.3)	2514 (54.5)	3248 (61.4)	2869 (69.8)	
Marry, n (%)						< 0.001
Never married	3429 (18.0)	1073 (21.5)	875 (19)	891 (16.8)	590 (14.3)	
Married or Living with partner	11417 (60.1)	2670 (53.6)	2757 (59.8)	3324 (62.9)	2666 (64.8)	
Others	4152 (21.9)	1240 (24.9)	982 (21.3)	1073 (20.3)	857 (20.8)	
Family PIR, Median (IQR)	2.1 (1.1, 4.1)	1.6 (0.9, 3.0)	2.0 (1.1, 3.8)	2.4 (1.2, 4.5)	3.0 (1.5, 5.0)	< 0.001
Hypertension, n (%)	9050 (47.6)	2521 (50.6)	2098 (45.5)	2449 (46.3)	1982 (48.2)	< 0.001
Diabetes, n (%)	3401 (17.9)	1090 (21.9)	839 (18.2)	872 (16.5)	600 (14.6)	< 0.001
Hyperlipidemia, n (%)	14171 (74.6)	3796 (76.2)	3450 (74.8)	3891 (73.6)	3034 (73.8)	0.012
CKD, n (%)	3324 (17.5)	1044 (21)	780 (16.9)	848 (16)	652 (15.9)	< 0.001
UA, Median (IQR)	5.3 (4.4, 6.3)	5.5 (4.5, 6.5)	5.4 (4.4, 6.4)	5.3 (4.4, 6.3)	5.2 (4.3, 6.1)	< 0.001
Hyperuricemia, n (%)	3817 (20.1)	1198 (24)	959 (20.8)	998 (18.9)	662 (16.1)	< 0.001
Gout, n (%)	886 (4.7)	280 (5.6)	187 (4.1)	237 (4.5)	182 (4.4)	0.002
Energy, Median (IQR)	1923.4 (1479.1, 2475.5)	1509.0 (1151.0, 1926.2)	1892.0 (1508.2, 2342.0)	2137.2 (1683.0, 2712.6)	2270.5 (1780.5, 2880.5)	< 0.001

PIR, poverty income ratio.

CKD, chronic kidney disease.

UA, uric acid.

### 3.2 Association between OBS and UA

Linear regression was employed to assess the association between OBS and plasma UA. The data presented in **Table 2** reveals a negative correlation between OBS and plasma UA (*p* < 0.05). In the fully adjusted model (Model 2), the highest quartile of OBS exhibited a stronger negative association with UA levels compared to the lowest quartile of OBS (β=-0.31 (-0.36, -0.25), *p* < 0.001), and this association remained relatively stable across models. We further explored the impact of dietary OBS and lifestyle OBS on UA using linear regression models. In Model 2, both dietary OBS and lifestyle OBS in the highest quartile group (Q4) were significantly associated with decreased uric acid levels compared to the lowest quartile (Q1) (β=-0.18 (-0.24, -0.12), *p* < 0.001, β=-0.64 (-0.69, -0.59), *p* < 0.001, respectively) (**Table 2**). The trend test indicated a statistically significant downward trend (*p* for trend < 0.001). These findings underscore the inverse relationship between OBS and plasma UA, highlighting the potential role of oxidative balance in modulating UA levels.

**Table 2:** Beta coefficient (95% CI) for serum UA by OBS/dietary OBS/lifestyle OBS quartiles.

Variable	Crude model		Model 1		Model 2	
	Beta coefficient 95%CI	*P* value	Beta coefficient 95%CI	*P* value	Beta coefficient 95%CI	*P* value
OBS						
Q1	0 (Ref)		0 (Ref)		0 (Ref)	
Q2	-0.11 (-0.17, -0.05)	<0.001	-0.11 (-0.16, -0.06)	<0.001	-0.08 (-0.13, -0.03)	0.002
Q3	-0.21 (-0.26, -0.15)	<0.001	-0.2 (-0.25, -0.15)	<0.001	-0.16 (-0.21, -0.11)	<0.001
Q4	-0.34 (-0.39, -0.28)	<0.001	-0.37 (-0.43, -0.32)	<0.001	-0.31 (-0.36, -0.25)	<0.001
*P* for trend	-0.11 (-0.13, -0.09)	<0.001	-0.12 (-0.14, -0.1)	<0.001	-0.1 (-0.12, -0.08)	<0.001
Dietary OBS						
Q1	0 (Ref)		0 (Ref)		0 (Ref)	
Q2	-0.05 (-0.1, 0.01)	0.096	-0.05 (-0.1, 0)	0.052	-0.02 (-0.07, 0.04)	0.541
Q3	-0.15 (-0.21, -0.1)	<0.001	-0.14 (-0.19, -0.09)	<0.001	-0.09 (-0.14, -0.03)	0.002
Q4	-0.21 (-0.27, -0.16)	<0.001	-0.25 (-0.3, -0.2)	<0.001	-0.18 (-0.24, -0.12)	<0.001
*P* for trend	-0.07 (-0.09, -0.06)	<0.001	-0.08 (-0.1, -0.07)	<0.001	-0.06 (-0.08, -0.04)	<0.001
Lifestyle OBS						
Q1	0 (Ref)		0 (Ref)		0 (Ref)	
Q2	-0.29 (-0.34, -0.23)	<0.001	-0.28 (-0.33, -0.23)	<0.001	-0.25 (-0.3, -0.2)	<0.001
Q3	-0.49 (-0.55, -0.44)	<0.001	-0.48 (-0.53, -0.43)	<0.001	-0.41 (-0.46, -0.36)	<0.001
Q4	-0.69 (-0.75, -0.64)	<0.001	-0.74 (-0.79, -0.69)	<0.001	-0.64 (-0.69, -0.59)	<0.001
*P* for trend	-0.23 (-0.25, -0.21)	<0.001	-0.24 (-0.26, -0.23)	<0.001	-0.21 (-0.23, -0.19)	<0.001

Crude model: unadjusted model.

Model 1: adjusted for sex, age and race.

Model 2: adjusted for sex, age, race, education level, family PIR, marital status, hypertension, diabetes, hyperlipidemia, CKD and energy.

### 3.3 Association between OBS and hyperuricemia

As shown in **Table 3**, logistic regression analysis revealed a significant negative association between OBS and hyperuricemia. The risk of hyperuricemia gradually decreased with increasing quartiles in all models (*p* for trend <0.001). In the fully adjusted model (Model 2), participants in Q4 of OBS showed a lower risk of hyperuricemia compared to the reference Q1 (OR = 0.63 (0.55, 0.71), *p* < 0.001), and this association remained consistent across models. Furthermore, in Model 2, both dietary OBS and lifestyle OBS in Q4 compared with Q1 reduced the risk of hyperuricemia (OR = 0.76 (0.67, 0.9), *p* < 0.001; OR = 0.37 (0.33, 0.42), *p* < 0.001, respectively) (**Table 3**). The trend test indicated a statistically significant downward trend (*p* for trend < 0.001).

**Table 3:** Odds Ratio (95% CI) for hyperuricemia by OBS/dietary OBS/lifestyle OBS quartiles.

Variable	Crude model		Model 1		Model 2	
	OR 95%CI	*P* value	OR 95%CI	*P* value	OR 95%CI	*P* value
OBS						
Q1	1 (Ref)		1 (Ref)		1 (Ref)	
Q2	0.83 (0.75, 0.91)	<0.001	0.86 (0.78, 0.94)	0.002	0.88 (0.8, 0.98)	0.019
Q3	0.73 (0.67, 0.81)	<0.001	0.74 (0.68, 0.82)	<0.001	0.79 (0.71, 0.88)	<0.001
Q4	0.61 (0.55, 0.67)	<0.001	0.58 (0.52, 0.64)	<0.001	0.63 (0.55, 0.71)	<0.001
*P* for trend	0.85 (0.82, 0.88)	<0.001	0.84 (0.81, 0.87)	<0.001	0.86 (0.83, 0.9)	<0.001
Dietary OBS						
Q1	1 (Ref)		1 (Ref)		1 (Ref)	
Q2	0.9 (0.82, 0.99)	0.035	0.93 (0.85, 1.03)	0.158	0.97 (0.88, 1.08)	0.582
Q3	0.79 (0.71, 0.87)	<0.001	0.8 (0.72, 0.89)	<0.001	0.86 (0.77, 0.96)	0.007
Q4	0.73 (0.66, 0.81)	<0.001	0.69 (0.63, 0.77)	<0.001	0.76 (0.67, 0.86)	<0.001
*P* for trend	0.9 (0.87, 0.93)	<0.001	0.88 (0.86, 0.91)	<0.001	0.91 (0.88, 0.95)	<0.001
Life OBS						
Q1	1 (Ref)		1 (Ref)		1 (Ref)	
Q2	0.68 (0.62, 0.75)	<0.001	0.68 (0.62, 0.75)	<0.001	0.72 (0.65, 0.79)	<0.001
Q3	0.5 (0.46, 0.55)	<0.001	0.51 (0.46, 0.56)	<0.001	0.56 (0.51, 0.62)	<0.001
Q4	0.31 (0.28, 0.34)	<0.001	0.32 (0.28, 0.35)	<0.001	0.37 (0.33, 0.42)	<0.001
*P* for trend	0.69 (0.66, 0.71)	<0.001	0.69 (0.67, 0.72)	<0.001	0.73 (0.7, 0.75)	<0.001

Crude model: unadjusted model.

Model 1: adjusted for sex, age and race.

Model 2: adjusted for sex, age, race, education level, family PIR, marital status, hypertension, diabetes, hyperlipidemia, CKD and energy.

### 3.4 Association between OBS and gout

As shown in **Table 4**, logistic regression analysis found a significant negative association between OBS and gout. In the fully adjusted model (Model 2), OBS was associated with a lower risk of gout in Q4 compared to Q1 (OR = 0.72 (0.58, 0.91), *p* = 0.005), and this association maintained relative stability across models. The trend test indicated a statistically significant downward trend (*p* for trend=0.014). When examining lifestyle OBS, individuals in Q4 exhibited a decreased risk of gout (OR = 0.54 (0.43, 0.67), *p* < 0.001). However, the associations between dietary OBS and gout were not statistically significant in Q3 and Q4 compared to Q1 after adjusting for all confounders. Only participants in Q2 of dietary OBS exhibited a statistically significant decrease in the risk of gout compared to Q1 (OR=0.75 (0.62, 0.92), *p* = 0.006) in Model 2.

**Table 4:** Odds Ratio (95% CI) for gout by OBS/dietary OBS/lifestyle OBS quartiles.

Variable	Crude model		Model 1		Model 2	
	OR 95%CI	*P* value	OR 95%CI	*P* value	OR 95%CI	*P* value
OBS						
Q1	1 (Ref)		1 (Ref)		1 (Ref)	
Q2	0.71 (0.59, 0.86)	<0.001	0.74 (0.61, 0.9)	0.002	0.75 (0.61, 0.92)	0.005
Q3	0.79 (0.66, 0.94)	0.009	0.78 (0.65, 0.94)	0.008	0.82 (0.67, 1)	0.052
Q4	0.78 (0.64, 0.94)	0.01	0.67 (0.55, 0.82)	<0.001	0.72 (0.58, 0.91)	0.005
*P* for trend	0.93 (0.87, 0.99)	0.016	0.89 (0.83, 0.95)	<0.001	0.91 (0.85, 0.98)	0.014
Dietary OBS						
Q1	1 (Ref)		1 (Ref)		1 (Ref)	
Q2	0.72 (0.6, 0.87)	0.001	0.75 (0.62, 0.91)	0.003	0.75 (0.62, 0.92)	0.006
Q3	0.86 (0.71, 1.03)	0.099	0.86 (0.71, 1.04)	0.127	0.9 (0.73, 1.11)	0.332
Q4	0.88 (0.73, 1.05)	0.158	0.76 (0.63, 0.92)	0.005	0.8 (0.64, 1)	0.051
*P* for trend	0.97 (0.91, 1.03)	0.308	0.93 (0.87, 0.99)	0.019	0.95 (0.88, 1.02)	0.162
Lifestyle OBS						
Q1	1 (Ref)		1 (Ref)		1 (Ref)	
Q2	0.81 (0.68, 0.96)	0.016	0.75 (0.63, 0.9)	0.002	0.84 (0.71, 1.01)	0.065
Q3	0.64 (0.53, 0.76)	<0.001	0.61 (0.51, 0.74)	<0.001	0.71 (0.59, 0.87)	0.001
Q4	0.43 (0.35, 0.53)	<0.001	0.41 (0.33, 0.51)	<0.001	0.54 (0.43, 0.67)	<0.001
*P* for trend	0.77 (0.72, 0.81)	<0.001	0.76 (0.71, 0.81)	<0.001	0.82 (0.77, 0.88)	<0.001

Crude model: unadjusted model.

Model 1: adjusted for sex, age and race.

Model 2: adjusted for sex, age, race, education level, family PIR, marital status, hypertension, diabetes, hyperlipidemia, CKD and energy.

### 3.5 Subgroup analysis

**Figure 2:**
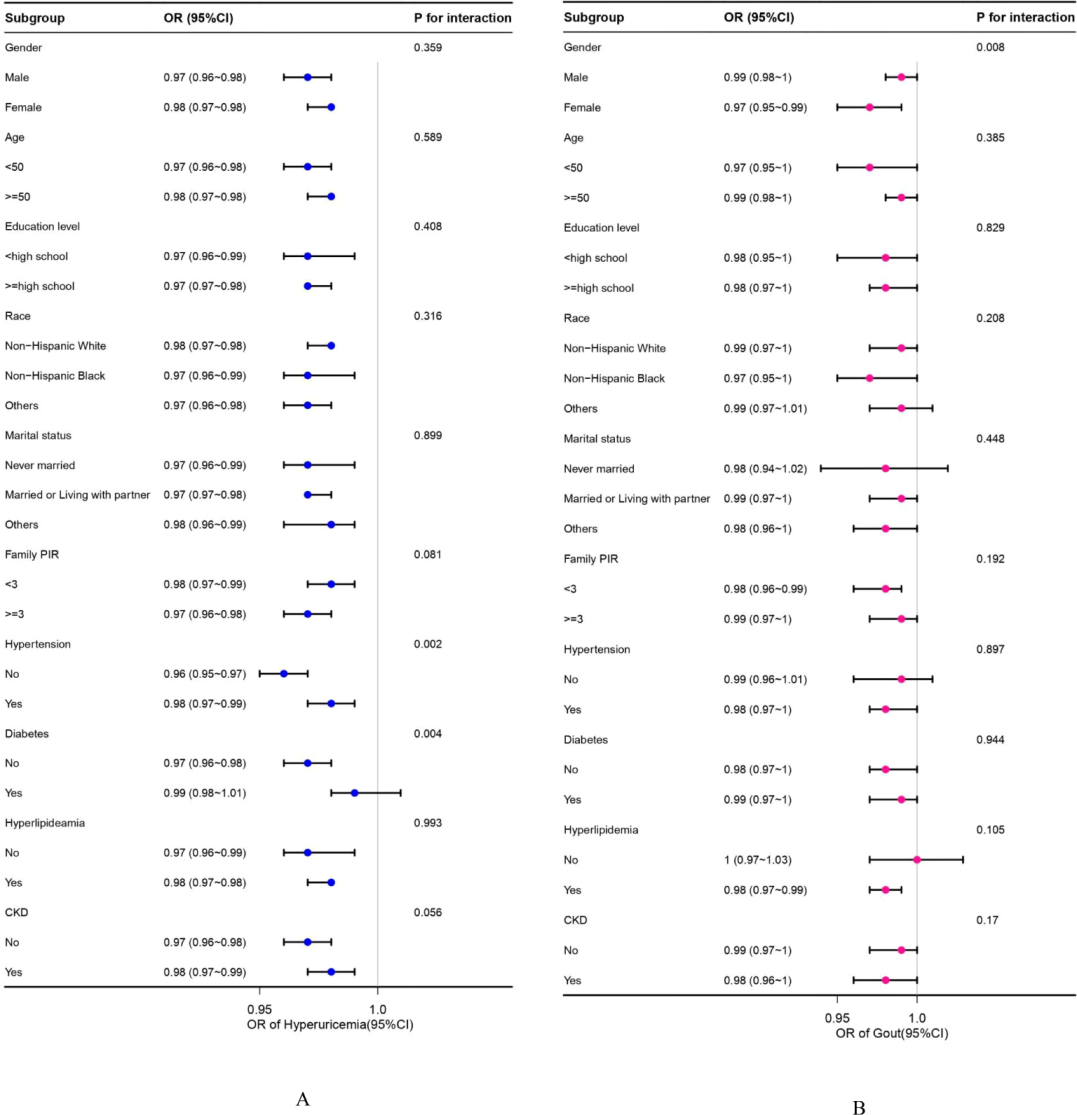
**(A, B)** Subgroup analyses between OBS and hyperuricemia/gout. **(A)** Hyperuricemia; **(B)** Gout. OR values are based on different population stratifications, estimated using a multivariable logistic regression model to assess the OR and 95% CI between OBS and hyperuricemia/gout. *P* for interaction values pertains to the significance of models considering interaction terms between OBS and various variables (sex, age, race, education level, family PIR, marital status, hypertension, diabetes, hyperlipidemia, and CKD) except for the stratification component itself. OBS, oxidative balance score; OR, odds ratio; CI, confidence intervals; CKD, chronic kidney disease.

**Figure 3:**
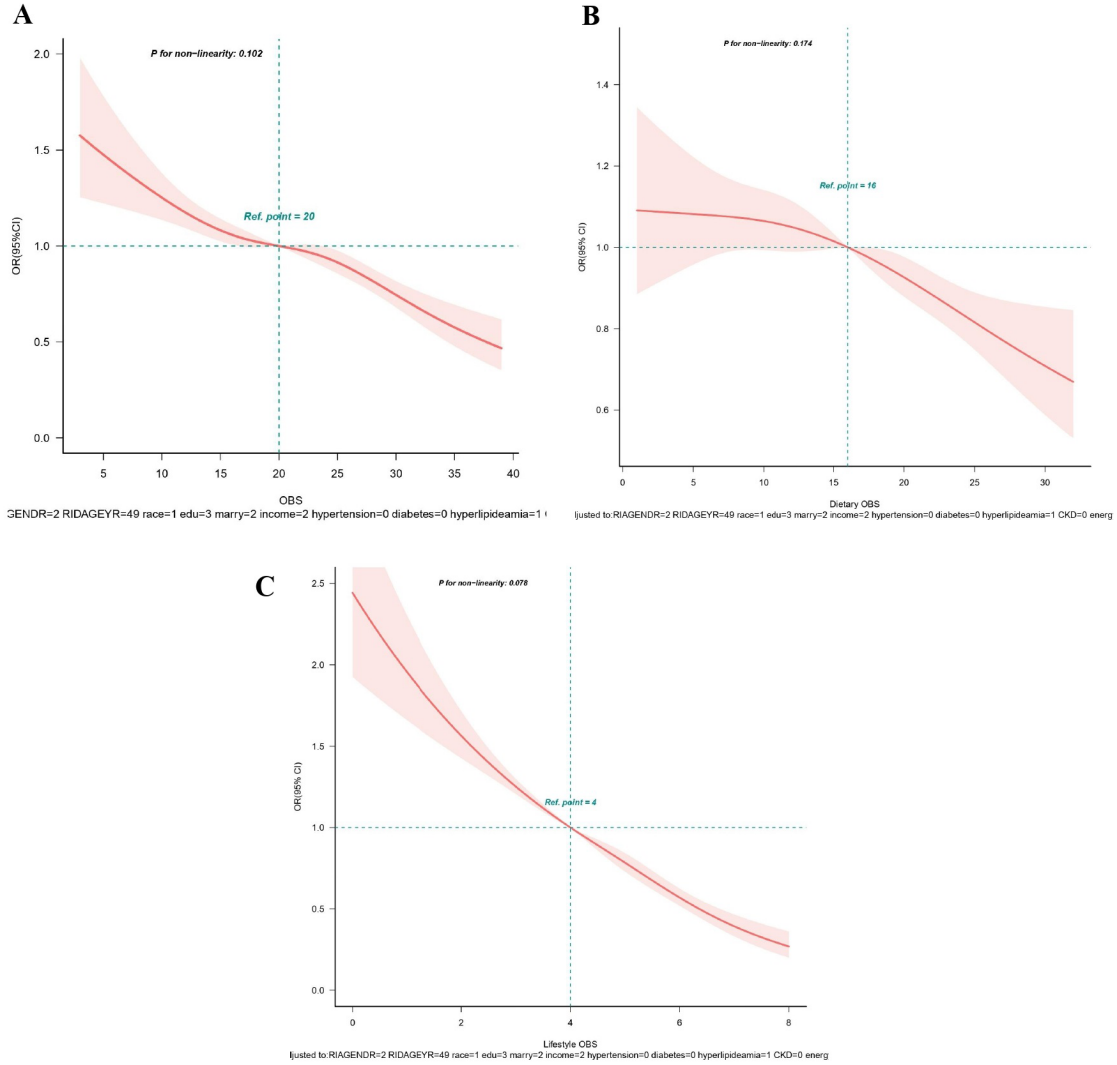
**(A-C)** Restricted spline curves for the associations between all OBS and hyperuricemia. **(A)** OBS; **(B)** Dietary OBS; **(C)** Lifestyle OBS. Red lines represent the OR and red transparent areas represent the 95% CI. OR (95% CI) were all adjusted according to Model 2. OBS, oxidative balance score; OR, odds ratio; CI, confidence intervals.

Subgroup analyses and interaction tests, stratified by sex, age, education level, race, family PIR, hypertension, diabetes, hyperlipidemia, and CKD, were conducted to identify potential variations across different populations. In hyperuricemia, as shown in **Figure 2**, statistical significance was found in hypertension and diabetes subgroups (*P* for interaction =0.002, *P* for interaction =0.004, respectively). The negative association effect of OBS with hyperuricemia was significantly greater in participants without hypertension (OR=0.96 (0.95, 0.97)) than in those with hypertension (OR = 0.98 (0.97, 0.99)). Similarly, the negative association effect of OBS was significantly greater in participants without diabetes (OR = 0.97 (0.96, 0.98)) than in those with diabetes (OR = 0.99 (0.98, 1.01)). In the analysis of gout prevalence (**Figure 3**), statistical significance was found in gender subgroups (*P* for interaction =0.008). A considerably stronger negative association between OBS and the prevalence of gout was observed in females (OR=0.97 (0.95, 0.99)) compared to males (OR=0.99 (0.98, 1)). Despite some inconsistent effect values in certain subgroups, our findings suggest that the negative association between OBS and hyperuricemia/gout was consistently maintained across all subgroups.

### 3.6 RCS analysis

**Figure 4:**
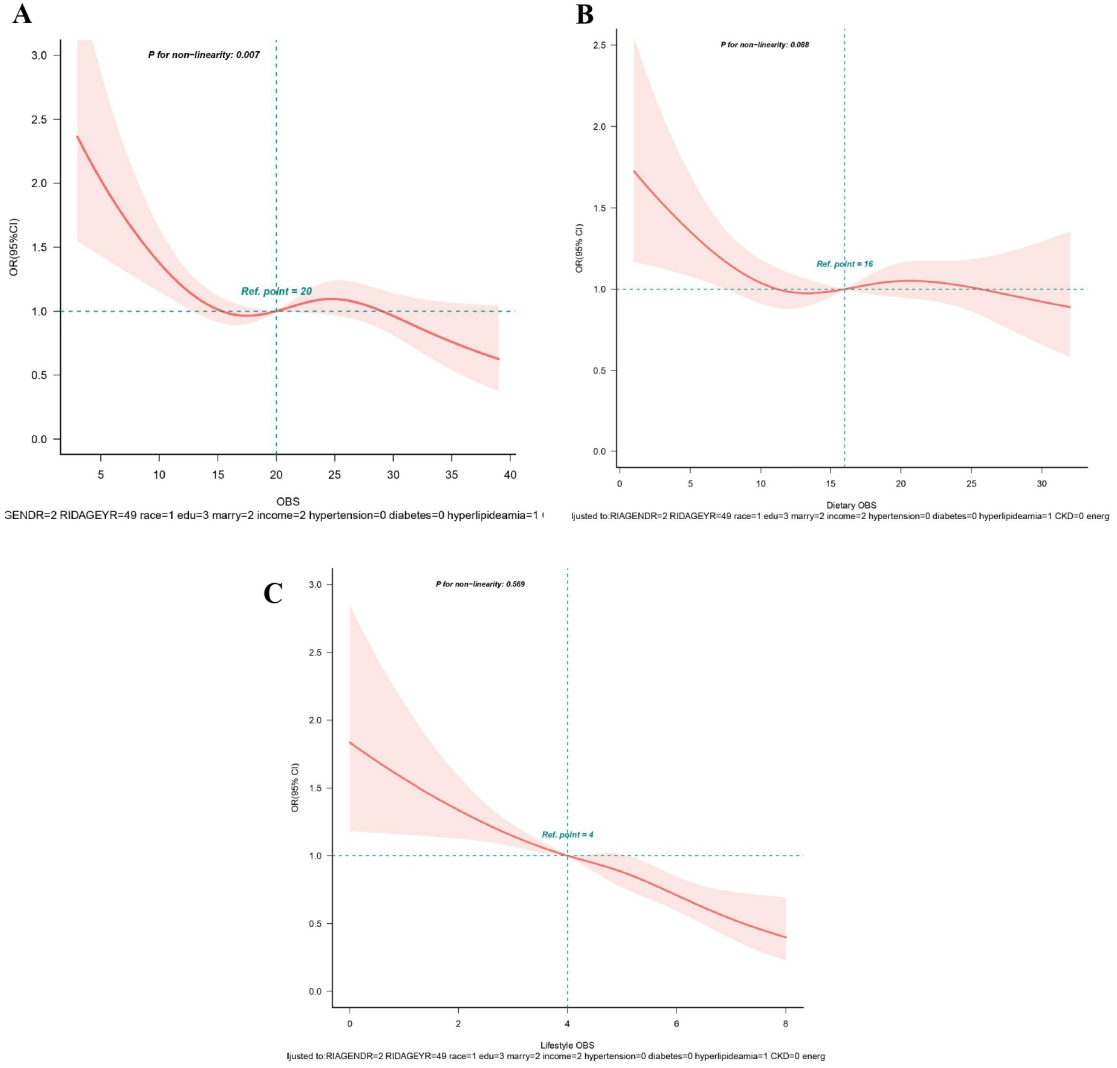
**(A-C)** Restricted spline curves for the associations between all OBS and gout. **(A)** OBS; **(B)** Dietary OBS; **(C)** Lifestyle OBS. Red lines represent the OR and red transparent areas represent the 95% CI. OR (95% CI) were all adjusted according to Model 2. OBS, oxidative balance score; OR, odds ratio; CI, confidence intervals.

From RCS analysis based on multivariable logistic regression adjusting for all covariates, a linear association was observed between OBS, dietary OBS, and lifestyle OBS and the prevalence of hyperuricemia (all *p* for non−linear > 0.05) (**Figures 3A–C**). A significant nonlinear relationship was identified between OBS and the risk of gout (*p* for non-linear < 0.05, **Figure 4A**). **Figures 4B, C** display that dietary OBS and lifestyle OBS were negatively and linearly associated with the risk of gout (*p* for non-linear > 0.05). These findings indicate that higher OBS is related to a lower prevalence of hyperuricemia and gout.

## 4 Discussion

To illuminate the relationship between OBS and hyperuricemia/gout, we conducted a cross-sectional analysis of 18998 individuals in the NHANES cohort. Our study found that total OBS was negatively associated with the levels of UA and the prevalence of hyperuricemia/gout. In addition, both dietary and lifestyle components exhibited independent associations with UA and hyperuricemia. Besides, the risk of gout was observed with higher OBS in lifestyle components. These associations remained significant even after adjusting for potential confounders, suggesting that increased exposure to antioxidants and decreased exposure to pro-oxidants, as indicated by a higher OBS, could potentially reduce the risk of hyperuricemia/gout. To the best of our knowledge, our research is the first comprehensive retrospective study to investigate the association between OBS and hyperuricemia/gout prevalence. It may be considered a good indicator of hyperuricemia/gout prevalence. These findings may provide some potential theoretical reference for the prevention of hyperuricemia/gout through oxidative stress.

The pathogenesis of hyperuricemia/gout is multifactorial and complex, involving a mix of genetic, environmental, and dietary factors (9). Mechanistically, oxidative stress is essentially due to an imbalance between pro-oxidants and antioxidants in the body, which is considered a major contributor to cellular damage and disease progression (23). Existing evidence indicates that UA has complex chemical and biological actions, featuring antioxidant properties with potential protective effects (2). However, it can also act as a pro-oxidant, contributing to the development of diseases such as hypertension, metabolic syndrome, and cardiovascular diseases (2). The exact role of UA in oxidative stress is not fully elucidated and may depend on its chemical microenvironment (2, 23). Increasing evidence suggests that some of the harmful intracellular effects induced by UA are mediated by oxidative stress. For instance, during the UA metabolism process, xanthine oxidoreductase (XOR) catalyzes the hydroxylation of hypoxanthine to xanthine, inducing the generation of ROS, subsequently resulting in an imbalance in redox signaling (24). Additionally, animal experiments have indicated that hyperuricemia-induced renal oxidative stress promotes mitochondrial dysfunction and reduced ATP levels in rats (25). Furthermore, a study conducted by Sánchez-Lozada et al. demonstrated that an elevated UA concentration exacerbates intracellular ROS production through the activation of nicotinamide adenine dinucleotide phosphate (NADPH) oxidase (26). Our research results indicate that a high OBS exerts a protective effect against the development of hyperuricemia/gout, aligning with the current understanding of the role of oxidative stress in the pathogenesis of hyperuricemia/gout.

A longitudinal study has revealed that individuals with high genetic risk, who adhere to a healthy diet and lifestyle, experience a 40% reduction in the risk of developing hyperuricemia compared to those with unhealthy dietary and living habits (27). Similarly, maintaining a healthy diet and lifestyle is associated with a nearly one-third reduction in the risk of gout related to genetic factors (28). Regarding dietary factors, some studies propose that supplementing dietary antioxidants, such as dietary fiber (29), vitamin B2 (30), vitamin E (31), vitamin C (32), folate (33, 34), carotenoids (35), calcium (30), and zinc (34), may contribute to lower UA levels. Conversely, pro-oxidants, exemplified by fat (30) and iron (36) intake, are linked to elevated UA levels. In our study, the OBS incorporates various dietary and lifestyle factors to comprehensively assess an individual’s antioxidant and pro-oxidant status. The findings indicate that certain specific antioxidants within the OBS are significantly associated with a reduced risk of hyperuricemia and gout. Dietary fiber plays an important role in OBS. An experimental study has demonstrated that dietary fiber can reduce oxidative stress and inflammatory responses in hyperuricemic mice by regulating the Toll-like receptor 4/NF-κB signaling pathway. Additionally, dietary fiber shows potential in alleviating hyperuricemic by modulating the gut microbiota and metabolites (37). Vitamin E is also a potent antioxidant. It protects cell membranes from oxidative damage by inhibiting free radical production and reducing oxidative stress. Research has shown that higher vitamin E intake is associated with a lower risk of hyperuricemia (31). This suggests that vitamin E may mitigate the negative impact of oxidative stress on uric acid metabolism, thereby lowering the risk of hyperuricemia. Lifestyle factors also play a significant role, wherein an elevated body mass index (BMI) increases the risk of hyperuricemia, while physical activity lowers the risk (38). A study (39) has indicated that men with a BMI over 27.5 kg/m² have a 16-fold higher risk of gout compared to those with a BMI below 20 kg/m², and a 4-fold higher risk compared to those with a BMI under 25 kg/m². The impact of alcohol consumption on hyperuricemia/gout remains controversial (40), with some studies suggesting that moderate wine consumption may enhance endogenous UA clearance and prevent gout attacks, attributed to the presence of antioxidants and phytoestrogens in wine. In contrast, others argue occasional alcohol intake is associated with increased UA secretion and gout attacks (40). Considering the pro-oxidant nature of alcohol, in conjunction with our research findings, and taking into account other health issues associated with alcohol, we recommend limiting alcohol intake, especially for individuals with gout or those at risk. Interestingly, certain studies have reported that smokers exhibit significantly lower serum UA levels. This is attributed to prolonged exposure to cigarette smoke, which leads to a reduction in the production of endogenous UA with antioxidant defense capabilities (41). However, conflicting findings have been reported, suggesting higher UA levels in smokers (42).

Recognizing the potential antagonistic and synergistic effects of pro-oxidants or antioxidants, we employed the OBS as a comprehensive assessment of individual oxidative balance for the first time to study its impact on hyperuricemia/gout. Our research indicated that the OBS in the non-hyperuricemia/gout group was markedly higher than that in the hyperuricemia/gout group. Notably, we found that lifestyle OBS was more effective in reducing the risk of hyperuricemia compared to dietary OBS. According to NHANES III data (43), 44%, 9%, and 8% of the risk of hyperuricemia in the general adult population may be attributed to obesity, unhealthy diet, and alcohol consumption, respectively. A study suggests that dietary habits exert a greater impact on the likelihood of developing hyperuricemia in males, while lifestyle factors related to physical activity have a more significant impact on females (44). Surprisingly, an unexpected finding in our study was a seemingly insignificant correlation between dietary OBS and gout. The underlying mechanism remains unclear, possibly because gout is a multifactorial metabolic disease, and its pathogenesis should not rely solely on hyperuricemia or monosodium urate (MSU) crystal formation (45). However, a study using NHANES data indicated a negative correlation between a comprehensive dietary antioxidant index(CDAI), evaluated with zinc, selenium, carotenoids, and vitamins A, C, and E, and the prevalence of gout in U.S. adults (46). Therefore, future high-quality prospective studies are needed to further explore the relevant mechanisms.

In the subgroup analysis, our study indicated a significant impact of diabetes and hypertension on the relationship between OBS and hyperuricemia. A prospective investigation revealed an elevated risk of developing hyperuricemia in individuals with diabetes or hypertension at baseline (47). The potential influences of insulin resistance and renal sodium handling on the renal clearance rate of UA might contribute to explaining the increased prevalence of hyperuricemia in individuals with diabetes and hypertension (48). Consequently, we speculate that the protective effect of OBS against hyperuricemia may not be as pronounced in participants with these chronic diseases. In addition, our research suggested a notable gender influence on the correlation between OBS and gout. Specifically, in females, there is a significantly stronger negative correlation between OBS and the incidence of gout. It may be attributed to the differing roles of sex hormones in oxidative stress and uric acid metabolism. Estrogen, which is more prevalent in females, has been shown to have antioxidant properties that can enhance the body’s defense against oxidative stress. This hormone may reduce the production of ROS and increase the activity of antioxidant enzymes, thereby lowering oxidative damage and potentially reducing uric acid levels (49). A study has demonstrated that postmenopausal hormone therapy, whether estrogen alone or in combination with progestin, moderately reduces the risk of gout (50). In contrast, androgens, which are more prevalent in males, have been associated with increased oxidative stress and higher uric acid levels (50). These differences in hormone profiles could explain why females might experience a more pronounced protective effect from a high OBS, leading to a stronger association with reduced gout risk. Future research should explore whether different antioxidant interventions differ in effectiveness between men and women. Specifically, studies could investigate if tailored antioxidant supplementation or lifestyle modifications based on hormonal profiles could optimize the reduction of uric acid levels and prevent gout more effectively in each gender.

This study suggests that the OBS may be a valuable tool for managing hyperuricemia and gout. Clinicians could use OBS in dietary and lifestyle counseling by encouraging patients to increase antioxidant-rich foods, such as fruits and vegetables, while reducing pro-oxidative factors like processed foods, potentially lowering the risk of these conditions. However, implementing OBS-based interventions in clinical practice presents challenges. Calculating OBS requires detailed dietary and lifestyle information, which can be difficult to collect and interpret. Additionally, patients’ adherence may also vary, especially with significant lifestyle changes. Integrating OBS into clinical workflows may require additional training for healthcare providers and the development of supportive tools.

While antioxidants generally protect against oxidative stress, certain antioxidants may exhibit pro-oxidative effects at high doses, known as the “antioxidant paradox.” For example, high doses of vitamin C may generate ROS instead of neutralizing them (51). Similarly, high concentrations of synthetic vitamin E can exacerbate oxidative stress (52). The threshold at which antioxidants shift from protective to harmful effects varies depending on factors such as the specific antioxidant, the presence of metal ions, and the redox status of the cellular environment. These threshold effects are critical for interpreting our study’s findings. The OBS reflects the balance between pro-oxidative and antioxidative factors, with higher scores suggesting a more favorable oxidative balance. However, at high doses, antioxidants may become pro-oxidative, indicating this balance is not linear. Individuals with very high antioxidant intake may experience diminished or even adverse effects, which could complicate the relationship between OBS and disease risk. While our study supports the general protective role of antioxidants, future research should explore dose-dependent effects, identify conditions where antioxidants become pro-oxidative, and determine optimal intake levels to maximize benefits and minimize risks.

This study employed a nationally representative population-based investigation, ensuring the universality and representativeness of our findings. Additionally, we incorporated potential covariates based on previous research, significantly reducing the impact of confounding factors. Finally, the robustness and stability of our study results were underscored through stratified analysis and sensitivity analysis. However, inherent limitations do exist in our study. Firstly, due to its cross-sectional nature, establishing causation is not feasible. Secondly, although averaging the data from two 24-hour dietary recalls enhanced representativeness, it may not fully capture long-term dietary habits, considering potential recall bias and the limited variety of foods considered. Thirdly, the study did not account for potential threshold effects of antioxidants, as some antioxidants have demonstrated potential pro-oxidative activity at high doses or under specific conditions. Therefore, the results obtained are merely the outcome of statistical analysis, and future prospective research is needed to address these limitations and provide further insights.

## 5 Conclusion

In conclusion, this nationally representative cross-sectional study revealed that a higher OBS was associated with a decreased risk of developing hyperuricemia/gout. The finding underscores the importance of adhering to an antioxidant-rich diet and lifestyle, contributing to the prevention and treatment of hyperuricemia/gout. However, further research is necessary in future studies to investigate the causal relationship and precise mechanisms underlying the association between OBS and hyperuricemia/gout.

## Data Availability

The original contributions presented in the study are included in the article/**Supplementary Material**. Further inquiries can be directed to the corresponding authors.
