# Supplementary material for: Association of oxidative balance score with hyperuricemia and gout: NHANES 2009-2018
Source: Front Endocrinol (Lausanne). 2024 Nov 4;15:1402369. doi: 10.3389/fendo.2024.1402369 (PMC11570265; doi:10.3389/fendo.2024.1402369)
Supplement: Supplementary file 1 [file Table1.docx]

Supplementary Table 1 Components of the oxidative balance score

| OBS components | Property | Male | | | Female | | |
| --- | --- | --- | --- | --- | --- | --- | --- |
|  |  | 0 | 1 | 2 | 0 | 1 | 2 |
| Dietary OBS components | | | | | | | |
| Dietary fiber (g/d) | A | <13.35 | 13.35-21.30 | ≥21.30 | <11.35 | 11.35-17.45 | ≥17.45 |
| Carotene (RE/d) | A | <99.37 | 99.37-322.31 | ≥322.31 | <104.88 | 104.88-355.79 | ≥355.79 |
| Riboflavin (mg/d) | A | <1.90 | 1.90-3.09 | ≥3.09 | <1.52 | 1.52-2.53 | ≥2.53 |
| Niacin (mg/d) | A | <25.36 | 25.36-38.82 | ≥38.82 | <18.65 | 18.65-29.54 | ≥29.54 |
| Vitamin B_6_ (mg/d) | A | <1.96 | 1.96-3.45 | ≥3.45 | <1.52 | 1.52-2.81 | ≥2.81 |
| Total folate (mcg/d) | A | <371.50 | 371.50-699.50 | ≥699.50 | <303.50 | 303.50-669.50 | ≥669.50 |
| Vitamin B_12_ (mcg/d) | A | <4.60 | 4.60-11.27 | ≥11.27 | <3.54 | 3.54-9.22 | ≥9.22 |
| Vitamin C (mg/d) | A | <54.25 | 54.25-136.55 | ≥136.55 | <53.60 | 53.60-132.20 | ≥132.20 |
| Vitamin E (ATE) (mg/d) | A | <6.25 | 6.25-10.04 | ≥1.04 | <5.26 | 5.26-8.44 | ≥8.44 |
| Calcium (mg/d) | A | <798.00 | 798.00-1251.50 | ≥1251.50 | <718.50 | 718.50-1137.00 | ≥1137.00 |
| Magnesium (mg/d) | A | <270.00 | 270.00-388.00 | ≥388.00 | <219.00 | 219.00-311.50 | ≥311.50 |
| Zinc (mg/d) | A | <10.79 | 10.79-17.56 | ≥17.56 | <8.05 | 8.05-13.70 | ≥13.70 |
| Copper (mg/d) | A | <1.09 | 1.09-1.68 | ≥1.68 | <0.90 | 0.90-1.41 | ≥1.41 |
| Selenium (mcg/d) | A | <110.65 | 110.65-162.70 | ≥162.70 | <80.75 | 80.75-118.10 | ≥118.10 |
| Total fat (g/d) | P | ≥100.91 | 68.41-100.91 | <68.41 | ≥76.47 | 52.25-76.47 | <52.25 |
| Iron (mg/d) | P | ≥19.42 | 12.92-19.42 | <12.92 | ≥16.92 | 10.43-16.92 | <10.43 |
| Lifestyle OBS components | | | | |  |  |  |
| Physical activity (MET-minute/week) | A | <680.00 | 680.00-3840.00 | ≥3840.00 | <200.00 | 200.00-1760.00 | ≥1760.00 |
| Alcohol (g/d) | P | ≥30 | 0-30 | None | ≥15 | 0-15 | None |
| Body mass index (kg/m^2^) | P | ≥30.50 | 26.00-30.50 | <26.00 | ≥32.00 | 25.80-32.00 | <25.80 |
| Cotinine (ng/mL) | P | ≥0.68 | 0.02-0.68 | <0.02 | ≥0.09 | 0.01-0.09 | <0.01 |

OBS: oxidative balance score; A: antioxidant; P: prooxidant; RE: retinol equivalent; ATE: alpha-tocopherol equivalent; MET: metabolic equivalent.
